# The role of imaging in the management of sinonasal mucoceles

**DOI:** 10.11604/pamj.2019.34.3.18677

**Published:** 2019-09-02

**Authors:** Rachida Bouatay, Lamia Aouf, Badii Hmida, Amel El Korbi, Naourez Kolsi, Khaled Harrathi, Jamel Koubaa

**Affiliations:** 1 ENT Department at Fattouma Bourguiba Hospital, Monastir, Tunisie; 2 Radiology Department at Fattouma Bourguiba Hospital, Monastir, Tunisie

**Keywords:** Mucoceles, paranasal sinuses, imaging

## Abstract

Mucoceles are slow-growing paranasal sinus cystic masses whose clinical presentation varies according to the affected sinus. Diagnosis is often radiological, based essentially on CT scan. The aim of this work was to study the radiologic characteristics of mucoceles on CT scan and MRI. We conducted a retrospective study of patients with mucoceles explored by imaging and operated on in our department. In our series, fronto-ethmoidal sinuses were the most frequently affected (81%). Facial scan confirmed the diagnosis in the majority of cases. Magnetic resonance imaging (MRI) was performed in 4 cases. Eleven patients were operated on by endonasal approach, three by external approach and one by combined surgical approach. Recurrence was observed in two patients after an average delay of 24 months. CT scan is considered the method of choice in the investigation of mucoceles. MRI is indicated in some cases to assess any orbital or intracranial extension.

## Introduction

Mucoceles are benign pseudo cystic, expansive, slow-growing paranasal sinus lesions, developed at the expense of sinus cavities and lined with an epithelium generally containing aseptic mucus [1,2]. Despite their benign histological nature, mucoceles can be potentially aggressive towards neighboring structures (orbit and encephalon) [3]. Clinical symptoms vary according to the involved sinus. The diagnosis is often radiological, based essentially on CT scan [4]. Surgical excision is the treatment of choice for mucoceles. It consists of a marsupialization of the lesion with nasal sinus drainage [3]. The objectives of our study were as follows: to illustrate the radiological characteristics of nasal sinus mucoceles and to study the contribution of imaging in determining their etiology and in assessing their extension.

## Methods

We retrospectively analyzed the medical records of all patients whose were clinically and radiologically diagnosed with nasal sinus mucoceles and subsequently operated on in the ENT and Cervicofacial Surgery Department of Fattouma Bourguiba University Hospital in Monastir between January 2000 and December 2016.

## Results

**Figure 1:**
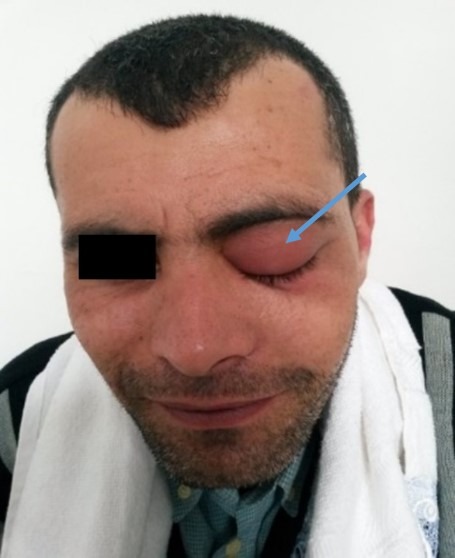
Left unilateral exophthalmia secondary to fronto-ethmoid mucocele

**Figure 2:**
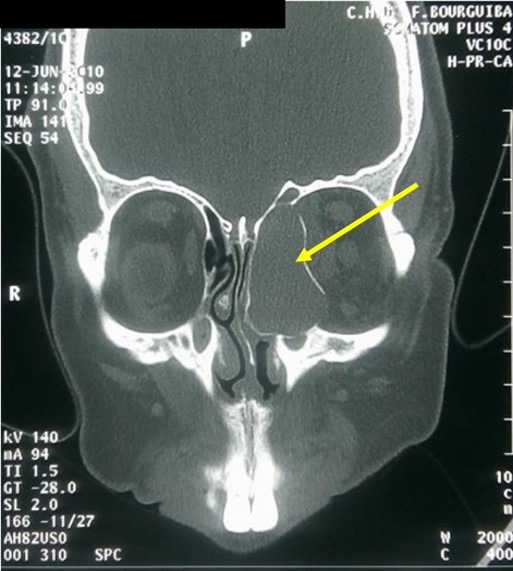
CT scan of the facial mass in the bony window, coronal section: left ethmoid mucocele with orbital extension (thinning of the bony walls and rupture in places)

**Figure 3:**
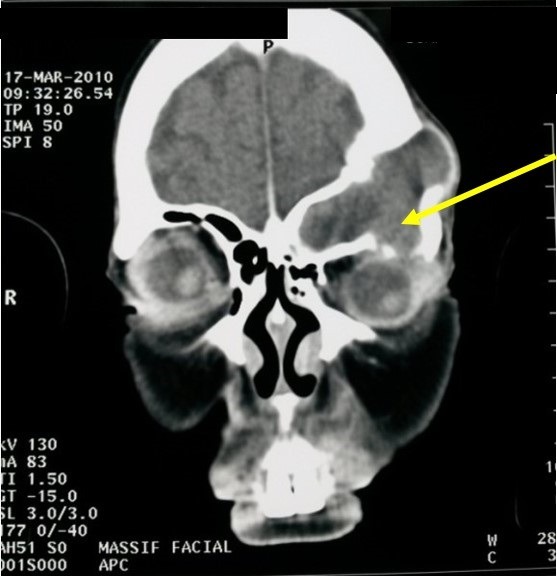
Facial CT scan in frontal section: left frontal mucocele responsible for a lysis in the left roof of the orbit

**Figure 4:**
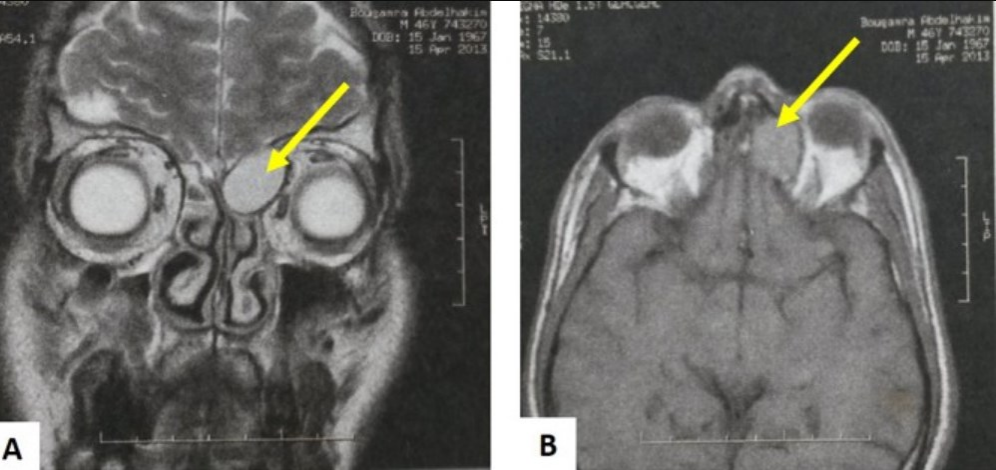
MRI of the facial area: in T1 axial section (A) and T2 frontal section (B): showing a well-defined left ethmoid cystic formation in discrete hyper-signal T1, hyper-T2 signal with intra orbital extension

Our study included 16 patients with nasal sinus mucoceles. The patients' mean age was 47 years (extremes: 15 and 83 years). The sex ratio was 1.14. Two patients (13%) had a history of rhino-sinus surgery (for nasal-sinus polyposis). Two patients reported a history of craniofacial trauma. One patient had chronic non-operated rhinosinusitis. The average consultation time was 16 months (1 month-84 months). The circumstances of discovery were varied. The essential mode of revelation was exophthalmia in 50% of cases (Figure 1). Rhinological signs were present in 13 patients (81%), and were dominated by nasal obstruction (6 cases). Nasal endoscopy revealed swelling of the anterior ethmoid in 2 cases, a middle meatus with a regular mass covered with a normal nasal mucosa in one case and with purulent secretions in 7 cases. Fifteen patients underwent a CT scan which substantiated the presence of a mucocele in all of them. In half of the cases, the mucocele presented as a well-defined cystic formation, homogeneous, hypodense or sometimes spontaneously hyperdense and whose contents do not take the contrast medium after iodine injection (Figure 2). These formations were expansive with thinning of bony walls. Mucocele was present at the level of the fronto-ethmoidal complex in 13 cases (81%), in the maxillary sinus in 2 cases (13%) and in the sphenoidal sinus in 1 case (6%). It was bilateral in one case. Bone lysis was found in all patients (Table 1); with the lamina papyracea, found in 6 cases (38%), being the most affected bone (Figure 3). The CT scan enabled us to objectify an intra-orbital extension in 9 cases: 7 cases of fronto-ethmoid mucoceles, one case of sphenoidal mucocele and one case of maxillary mucocele. No cases of endocranial extension were noted. It also revealed other associated abnormalities, such as bilateral maxillary sinus atelectasis associated with left frontal mucocele in one case, pansinusitis associated with ethmoid mucoceles in two cases, and concha bullosa of left fronto-ethmoid mucocele in one case, and Sino nasal polyposis in a case of left frontal mucocele. MRI was performed in 4 patients to assess the relationship of the mucocele with meninges and orbital structures. Endo-orbital extension was noted in 7 cases of fronto-ethmoid mucoceles, in one case of sphenoidal mucocele and in one case of maxillary mucocele (Figure 4). There was no case of endocranial extension. The signal of the mucocele was variable. In fact, it presented a hypo signal T1 in 2 cases (13%), a high signal in one case and T1 signal in one case. With T2, the mucocele presented a hyper signal in all four cases. All our patients were operated on and we performed: a large endonasal marsupialization in 12 patients; an external marsupialization in 3 cases (lateral frontal mucoceles); in 1 case, the endonasal approach was associated with an external approach. The mean follow-up was 5 years (range, 7 months to 10 years). The evolution was favorable in 14 cases; recurrence occurred in 2cases (a case of frontal mucocele initially operated by external approach and a case of ethmoid mucocele initially operated on by the endonasal approach). These two cases of recurrence were further managed by a combined surgical approach.

**Table 1:** Bone lysis according to the location of the mucocele

Bone lysis	Number of cases	Location of the mucocele				
		Frontal	E	FE	M	S
lamina papyracea	6 cases	1	1	3	-	1
Internal table of frontal sinus	2 cases	2	-	-	-	-
External table of frontal sinus	3 cases	3	-	-	-	-
Roof of the orbit	1 case	-	-	1	-	-
Postero-superior wall of sphenoid sinus	1 case	-	-	-	-	1

F: Frontal, E: Ethmoidal, FE: Fronto-Ethmoidal, M: Maxillary, S: Sphenoid

## Discussion

Mucocele is a benign and expansive pseudo cystic slow-growing paranasal sinus formation, developed at the expense of sinus cavities and lined with an epithelium generally containing aseptic mucus [1,2]. The total number of mucoceles described is increasing, due to the increasing incidence of secondary mucoceles, accounting for up to 60% of cases [4]. Sinus mucoceles occur most often in the third and fourth decades of life with a slight male predominance [3]. The average age of patients in our series was 47 years with extremes ranging from 15 years to 83 years. The sex ratio was 1.14. The onset of clinical symptoms is usually delayed, which explains some accidental radiological findings. Paranasal sinus mucoceles can present with a multitude of different symptoms according to the location of the mucocele and its volume [3]. In our series, the time of evolution of the disease ranged from 1 to 84 months, with an average delay of 16 months. Symptoms can be ophthalmological, neurological, rhinological, or aesthetic. They may also occur in a severe form, during superinfection (pyomucocele) or intraorbital rupture or at the anterior skull level [3,5]. Nasal endoscopy assesses surgical accessibility and eliminates associated naso-sinus pathology that should always be sought, even if it is rarely found [3]. Imaging plays a major role in the diagnostic and even in the management of mucoceles [6]. Facial scan is the first-line examination to be performed in case of clinical or endoscopic suspicion of mucocele [7]. The presence of an open expansive convex opacity without air developed at the expense of a sinus cavity with a homogeneous mucosal content (10 to 18 HU) is sufficient for a positive diagnosis [2]. The density of the mucocele depends on the degree of hydration; spontaneously hypodense or isodense, the old forms can appear hyperdense [3]. The density is not modified by iodine injection but a fine peripheral enhancement, corresponding to an enhancement of the mucosa, can be observed [8]. A hypodense aspect was found in half of our patients. CT is the most effective technique to assess the impact of mucocele on the bony walls, which are initially repressed and then lysed secondarily; bone condenses are rare, indicating a slow growth rate [4]. In our series, bone lysis was present in all cases. In case of meningeal dehiscence, the difference in density is not sufficient enough to differentiate a mucocele from an adjacent cerebral parenchyma and the injected sections enhancing the dura mater are of an interesting contribution [6].

The endo-orbital extension is often extraconic, On the CT it can be translated by a repression of the eyeball downwards, a repression of the internal and/or oblique rectus muscle. Sometimes, extrinsic compression of the lacrimal ducts is observed due to the mucocele protruding from the ethmoidal bulla [9]. In our series, an orbital extension was found in 9 patients. CT is the best method for locating mucoceles [2]. Fronto-ethmoid mucoceles are the most common locations [2,10]. In accordance with this, Fronto-ethmoid mucoceles represent 81% of the cases in our series. CT also makes it possible to search for local etiological factors [3] and to eliminate concha bullosa, high septal deflection, ethmoidal inflammation and any other acquired or congenital pathologies of the middle meatus responsible for poor aeration [11]. In our series, the CT scan showed a bilateral concha bullosa with subtotal filling of the left maxillary sinus in a case of left fronto-ethmoid mucocele. MRI is indicated in case of diagnostic doubt between a mucocele and other tumoral or inflammatory lesions or in case of intraorbital or intracranial extension and in case of sphenoidal localization [7]. The intensity of the signal varies according to the protein content of secretions contained and whether there is infection [12]. Thus, the weighted signal in T1 and T2 changes with time. A recent mucocele with a low protein content will be T1 hyposignal and T2 hypersignal. On the other hand, as we age, the level of protein increases with a more intense signal in T1 and a hypointense in T2 [2]. A total absence of signal is thus possible, giving the false impression that the sinus is aerated; this highlights the importance of the scanner as the first diagnostic tool [4]. The injection of a gadolinium-based contrast agent results in peripheral linear enhancement without enhancing the mucocele content. A malignant tumor gives a heterogeneous and global enhancement [6]. MRI allows to specify the relationship of the mucocele with the sinus adjacent noble organs (dura mater, pituitary gland, optic nerves and cavernous lodges) and to guide therapeutic choices [8]. Thus, we understand the value and the wide use of this imaging tool in cases of fronto-ethmoid, sphenoidal or posterior ethmoid mucoceles [4]. The therapeutic strategy for mucoceles depends essentially on the location and extent of the lesions, determined by imaging data. There are essentially two primary approaches: the external one, and the endonasal endoscopic approach [13]. The principle of endoscopic surgery is based on marsupialization while respecting preserving the healthy mucosa and widening the natural drainage pathways [2,14].

It respects the functional properties of the rhino-sinus mucosa, thus limiting the complications, the morbidity and the duration of hospitalization associated with the external surgery [4]. However, external surgery still retains certain indications, represented by the limits of endonasal surgery [3,4,7]: mucocele of the lateral wall of the maxillary sinus is difficult to control even with lateral optics 70°, and is too small, making endonasal marsupialization insufficient, and carrying the risk of synechia and reccurrence in the short term; mucocele with cutaneous fistula, for which it is necessary to resect the fistulous path; mucocele associated with a tumoral pathology; lateral frontal involvement often requires an external (brow or coronal) or combined approach, in case of inaccessibility to the mucocelic pocket by endonasal Surgery, even after resection of the frontal sinus floor [13]. In these situations, a combination of external and endonasal approaches with the external approach kept to the minimum, or even an exclusive external approach, is justified [3]. Apart from complications, patients will be reviewed for endoscopy and MRI monitoring every 6 months during the first two years. In the long term, systematic monitoring consists in annual MRI exams [2]. Paranasal sinuses mucoceles may recur several years after surgical treatment. Recurrence rate varies from 3 to 35% according to authors [15]. As a result, regular long-term endoscopic follow-up is highly recommended to check for possible complications or recurrence requiring a new imaging or a surgical revision [3]. For our patients, recurrence was noted in 2cases (12.5%).

## Conclusion

Sinonasal mucoceles are relatively rare benign pseudocystic lesions, with insidious evolution. Clinic suspicion of a mucocele is confirmed by tomodensitometry. CT scan is the first-line examination to conduct in case of clinical or endoscopic suspicion of a mucocele. MRI is indicated in cases of diagnostic doubt between a mucocele and other tumoral or inflammatory lesions or in case of intraorbital or intracranial extension and sphenoidal location.

### What is known about this topic

- The increasing incidence of secondary sinonasal mucoceles;
- Despite their benign histological nature, sinonasal mucoceles can be potentially aggressive towards orbit and encephalon;
- The diagnosis is often radiological, based essentially on CT scan.

### What this study adds

- The aim of this work was to study the contribution of imaging in the management of sinonasal mucoceles (confirmation, etiology and in assessing their extension)
- Facial scan is the first-line examination to be performed in case of clinical or endoscopic suspicion of mucocele;
- MRI to assess the relationship of the mucocele with meninges and orbital structure. These two exams are complementary for the management of sinonasal mucoceles.

## Competing interests

The authors declare no competing interests.
